# PlcRa, a New Quorum-Sensing Regulator from *Bacillus cereus*, Plays a Role in Oxidative Stress Responses and Cysteine Metabolism in Stationary Phase

**DOI:** 10.1371/journal.pone.0051047

**Published:** 2012-12-11

**Authors:** Eugénie Huillet, Marcel H. Tempelaars, Gwenaëlle André-Leroux, Pagakrong Wanapaisan, Ludovic Bridoux, Samira Makhzami, Watanalai Panbangred, Isabelle Martin-Verstraete, Tjakko Abee, Didier Lereclus

**Affiliations:** 1 INRA, UMR1319 Micalis, Génétique microbienne et Environnement, Guyancourt, France; 2 Wageningen University, Laboratory of Food Microbiology, Wageningen, The Netherlands; 3 Institut Pasteur, Microbiologie Structurale, CNRS, UMR 3528, Paris, France; 4 Mahidol University, Department of Biotechnology, Faculty of Science, Bangkok, Thailand; 5 INRA, UMR GABI, ICE, Jouy-en-Josas, France; 6 Institut Pasteur, Laboratoire de Pathogénèse des Bactéries Anaérobies, Paris, France; 7 Univ. Paris Diderot, Sorbonne Paris Cité, Cellule Pasteur, Paris, France; Loyola University Medical Center, United States of America

## Abstract

We characterized a new quorum-sensing regulator, PlcRa, which is present in various members of the *B. cereus* group and identified a signaling heptapeptide for PlcRa activity: PapRa_7_. We demonstrated that PlcRa is a 3D structural paralog of PlcR using sequence analysis and homology modeling. A comparison of the transcriptomes at the onset of stationary phase of a Δ*plcRa* mutant and the wild-type *B. cereus* ATCC 14579 strain showed that 68 genes were upregulated and 49 genes were downregulated in the Δ*plcRa* mutant strain (>3-fold change). Genes involved in the cysteine metabolism (putative CymR regulon) were downregulated in the Δ*plcRa* mutant strain. We focused on the gene with the largest difference in expression level between the two conditions, which encoded -AbrB2- a new regulator of the AbrB family. We demonstrated that purified PlcRa bound specifically to the *abrB2* promoter in the presence of synthetic PapRa_7_, in an electrophoretic mobility shift assay. We further showed that the AbrB2 regulator controlled the expression of the *yrrT* operon involved in methionine to cysteine conversion. We found that the Δ*plcRa* mutant strain was more sensitive to hydrogen peroxide- and disulfide-induced stresses than the wild type. When cystine was added to the culture of the Δ*plcRa* mutant, challenged with hydrogen peroxide, growth inhibition was abolished. In conclusion, we identified a new RNPP transcriptional regulator in *B. cereus* that activated the oxidative stress response and cysteine metabolism in transition state cells.

## Introduction

The *Bacillus cereus* group includes well known spore-forming pathogens of mammals (*B. anthracis* and *B. cereus*) and insects (*B. thuringiensis*). *B. cereus* is frequently associated with food-borne infections causing gastroenteritis [1]. The capacity of *B. cereus* to sporulate allows this bacterium to resist the usual cleaning procedures used in the food industry, resulting in the presence of *B. cereus* in many raw and processed foods, such as rice, spices, milk, vegetables, meats and various desserts [1].

At the end of the vegetative growth phase, bacterial cells face a number of challenges, including a decrease in the nutrient content of their environment. Under these conditions, spore-forming bacteria may initiate sporulation, producing spores that can survive in unfavorable environmental conditions [2]. Bacteria make use of various strategies to cope with environmental changes during the transition between the vegetative and sporulation phases [3]. The production of degradative enzymes and antimicrobial compounds responsible for the lysis of targeted cells provides *Bacillus subtilis* with new nutrients [3]. In parallel, a general stress response may be activated during the transition phase, due to the accumulation of oxidative products and changes in the pH of the medium [4]. Cellular responses may be controlled by a range of sensors and activators, including two-component systems, quorum-sensing systems and other transcriptional regulators [3], [5].

Quorum sensing regulation appears to be a consequence of interbacterial communication by which bacteria of one or even different species sense about their current population density and react in a defined way to that information. These communication systems are based on the secretion and recognition of cell-cell signalling molecules, termed autoinducers [6]. The PlcR/PapR quorum sensing system is activated during the transition phase in most members of the *B. cereus* group [7]. This system controls the expression of genes encoding exported virulence factors, including degradative enzymes, enterotoxins and hemolysins [8]. PlcR is activated by binding to PapR, a signaling peptide produced as a propeptide under the control of PlcR. PapR undergoes extracellular processing, to generate an active heptapeptide [9], which is then re-imported into the bacterial cell via the oligopeptide permease system, OppABCDF [10]. Within the cell, PapR interacts with PlcR and the resulting complex binds PlcR target sites on DNA [11], resulting in the activation of the PlcR regulon, which contains 48 genes [7].

The structure of PlcR has been resolved. This molecule has a unique folding pattern, due to the presence of an HTH DNA-binding domain and a peptide-binding regulatory domain composed of five tetratricopeptide repeats (TPR) [12]. A TPR is a structural 34-amino acid repeat motif present in various eukaryotic and prokaryotic proteins. There may be from 3 to more than 16 tandem repeats [13]. Determination of the structure of PlcR resulted in the identification of a new family of central regulatory quorum sensors (the RNPP family) found exclusively in Gram-positive bacteria with a low G+C content [12]. These quorum sensors include NprR from *B. cereus* [14], PrgX from *Enteroccocus faecalis* [15] and RAP phosphatases from *B. subtilis* [16], [17]. All these RNPP regulators are activated through a secreted signaling peptide that interacts with the TPR activation domain.

In this work, we characterized PlcRa, a novel quorum-sensing type regulator of the RNPP family and identified PapRa_7,_ a new signaling heptapeptide. We constructed a Δ*plcRa* mutant strain from the *B. cereus* ATCC 14579 type strain [18] and identified PlcRa-controlled genes through a whole-genome microarray approach at the onset of stationary phase. We purified PlcRa protein and assessed the DNA binding activity of PlcRa using electrophoretic mobility shift assay. By combining *in silico* structural analysis, genetic and biochemical methods, the PlcRa activity in relation with the presence of PapRa_7_ was described.

## Results

### PlcRa is a 3D Structural Homolog of PlcR

**Figure 1:**
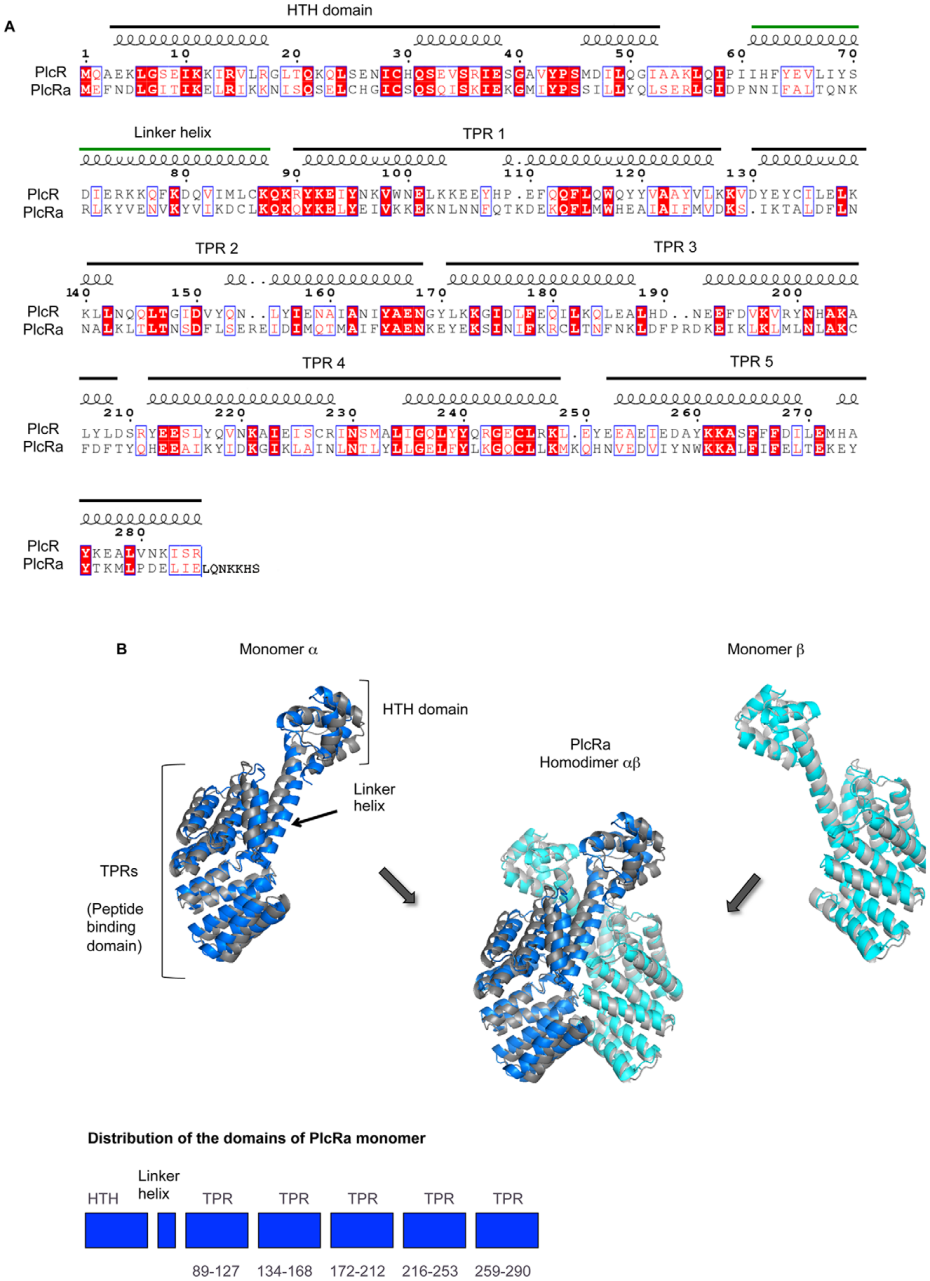
Homology modeling of PlcRa. A. Alignment of the sequences of *B. thuringiensis* 407 Cry^-^ PlcR chain A (pdb code: 2QFC_A) and ATCC 14579 *B. cereus* PlcRa. The highly conserved residues are indicated in red in blue boxes, the strictly conserved residues are indicated in white in red boxes. Helices were the only secondary structural elements found and are displayed with the predicted domains above the sequences. The numbers indicate positions relative to the PlcR sequence. B. Homology modeling of the PlcRa homodimer from the target structure of PlcR (pdb code: 2QFC). Chains A and B of PlcR are shown in gray; the modeled chains A and B of PlcRa are shown in blue and cyan, respectively. Below: distribution of the domains in each monomer of PlcRa: the HTH at the N-terminus followed by the linker helix, with the five TPR motifs at the C-terminus.

**Figure 2:**
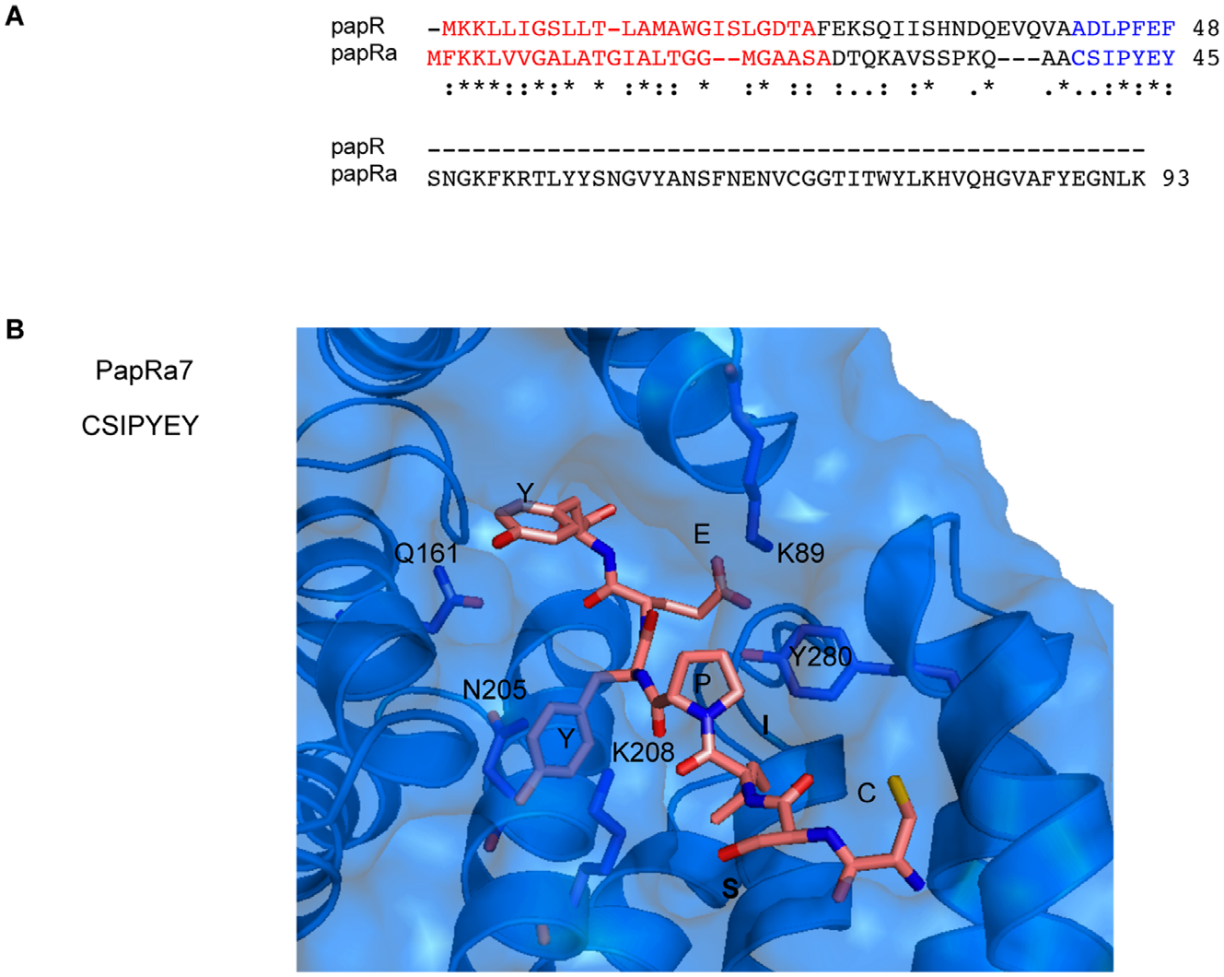
*In silico* analysis of PapRa_7,_ a new putative signal peptide. A. Sequence alignment of *B. thuringiensis* 407 Cry- PapR and *B. cereus* ATCC 14579 PapRa. The putative signal sequences are coloured in red and PapRa_7_ (CSIPYEY) and PapR_7_ (ADLPFEF) heptapeptides are highlighted in blue. B. *In silico* docking of PapRa_7_ in PlcRa TPR pocket. Close 90° view of the interaction of CSIPYEY with residues K89 (TPR1 motif), Q161 (TPR2 motif), N205 (TPR3 motif), K208 (TPR3 motif) & Y280 (TPR5 motif) (see Figure 1 for TPRs location). The residues N205 and K208 mediate peptide main chain binding by hydrogen bonds.

BLAST searches identified three PlcR paralogs encoded by the *B. cereus* ATCC 14579 genome: BC0988 (PlcRa), BC1158 (PlcRb, previously named PlcR2 in *B. anthracis* [19]) and BC2443 (PlcRc). These putative regulators display about 29% sequence identity and about 50% similarity to PlcR. The PlcRa, PlcRb and PlcRc proteins have high levels of overall sequence identity (85%). A small ORF, BC0989, encoding a putative peptide with a potential signal sequence is located upstream from the *plcRa* gene. We named this gene *papRa*. In contrast, the *plcRb* and *plcRc* genes are not associated with such genes. As all characterized RNPP regulators are activated by exported peptides with regulatory functions, we decided to focus on the *plcRa/papRa* locus. We describe here the analysis of *plcRa* in the *B. cereus* ATCC 14579 strain. Genome comparisons revealed genes for PlcRa (identity >94%) in *B. thuringiensis* BMB171, serovar chinensis CT-43, HD-789, HD-771 strains (4 out of 7 complete genomes) and *B. cereus* B4264 and G9842 strains (3, including ATCC 14579, out of 13 complete genomes) but no such gene was present in the genome of *B. anthracis* (data not shown). The 297-amino acid PlcRa protein displays 29% identity and 51% similarity to PlcR (Figure 1A). Given this high identity score over its entire sequence, PlcR is a relevant template for homology modeling [20]. We therefore constructed a homology model for PlcRa, based on the structure of the PlcR dimer solved at a resolution of 2.6 Å [12]. Each PlcR monomer displays a unique folding pattern, with an HTH domain at the N-terminus, followed by a linker helix connecting the HTH domain to the 5 TPR domains and serving as an anchoring platform for dimerization. The packing of the five TPR domains defines, for each monomer, a pocket that binds the PapR activator peptide [12]. The PlcRa homodimer model, composed of A and B chains, was constructed progressively, beginning with chain A, followed by the addition of chain B and ending with modeling of the dimer (Figure 1B). As expected, the homology model of the PlcRa homodimer was found to be highly helical. Each monomer has, at its N-terminus, an HTH domain followed by a linker helix of 27 residues (Figure 1A) that connects the HTH to the five TPRs and anchors the two monomers together (Figure 1B). Thus, the homology model of PlcRa constructed here is very similar to the X-ray structure of PlcR. By analogy with PlcR [12], we suggest that the five TPR motifs may be arranged similarly, to form a pocket that is responsible for peptide binding. The *papRa* gene encodes a 93-amino acid polypeptide, which is longer than PapR (45 amino acids). As described for PapR, a typical Gram-positive N-terminal signal peptide was identified for PapRa with SignalP program (Figure 2A) [21]. Interestingly, alignment of PapR and PapRa sequences showed similarity over a short segment corresponding to the PapR C-terminus including the heptapeptide (ADLPFEF), which is the physiological activator of PlcR (Figure 2A) [9]. Based on this sequence alignment, the CSIPYEY fragment -PapRa_7_ - was proposed as a consistent candidate for a signaling heptapeptide. We docked CSIPYEY into the dedicated pocket of PlcRa and minimized the energy of the complex with CHARMm [22]. This docking procedure showed that PapRa_7_ could fit into the PlcRa pocket formed by the five TPRs (Figure 2B). Overall, these homology modeling and docking analysis suggested that PlcRa is a 3D structural homolog of PlcR**.** For confirmation and characterization of PlcRa as a regulator, we first conducted a genetic analysis of the *plcRa* gene and searched for target genes using a comparative transcriptome analysis approach with the Δ*plcRa* strain.

### The Expression of *plcRa* is Activated at the Onset of the Stationary Growth Phase

**Figure 3:**
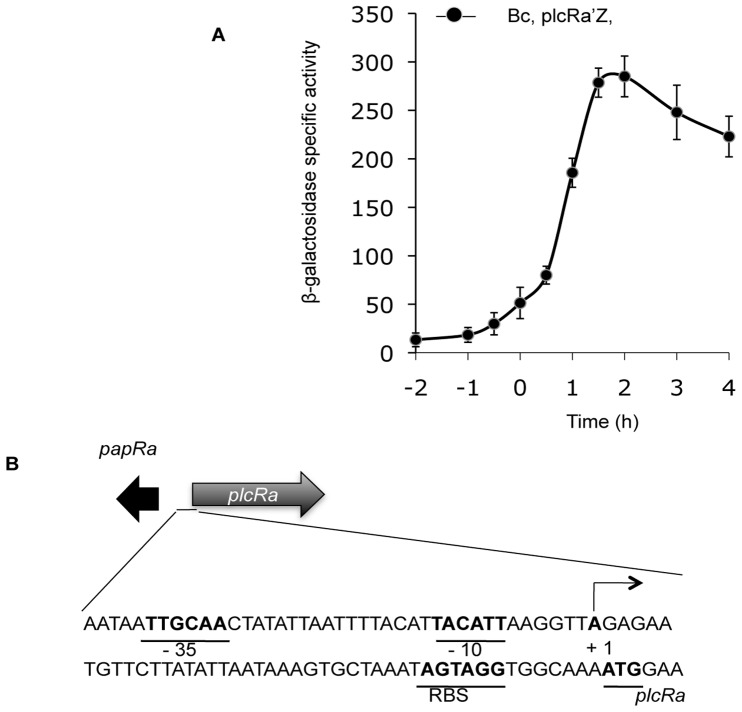
Analysis of *plcRa* expression. A. Kinetics of *plcRa* gene expression. Specific β-galactosidase activity (U/mg protein) of strain *B. cereus* ATCC 14579 harboring the transcriptional P*_plcRa’_-lacZ* fusion. Time zero corresponds to the onset of the stationary growth phase, and *t*_n_ is the number of hours before (–) or after time zero. The cells were grown at 37°C in LB medium. Error bars are shown. B. Determination of the transcriptional start site of *plcRa*. The 5′ RACE-PCR method was used to identify the transcriptional start site of *plcRa*. The start site (+1, in bold typeface) and the −10 and −35 putative promoter elements from the vegetative sigma factor are shown in bold typeface and underlined. The putative ribosome-binding site sequence and putative start codon of *plcRa* are shown in bold typeface and underlined.

We investigated the temporal regulation of *plcRa* gene expression, by constructing a P*_plcRa_’-lacZ* transcriptional fusion in the low-copy-number plasmid pHT304–18Z [23]. The P*_plcRa_’-lacZ* fusion was introduced into the *B. cereus* wild-type strain and β-galactosidase activity was measured from *t*_−2_ to *t*_4_ (time zero, *t*_0_, corresponds to the onset of the stationary growth phase, and *t*_n_ is the number of hours before (–) or after time zero) during growth in LB medium. Expression of the P*_plcRa_’-lacZ* fusion began at *t*_−1_ and increased rapidly from *t*_0.5_ to *t*_1.5_ (Figure 3A). These findings suggest that *plcRa* expression is transiently activated early in the stationary phase. We then constructed a *plcRa* mutant (Table 1) as described in Material and Methods. Expression of the P*_plcRa_’-lacZ* fusion was similar in the wild-type strain and in the *ΔplcRa* mutant strain during growth (data not shown), indicating that the *plcRa* gene is not autoregulated. The transcriptional start site identified by 5′RACE was located 46 bp upstream from the predicted start codon of *plcRa* (Figure 3B). The *plcRa* promoter region contains −10 and −35 DNA binding regions resembling those recognized by SigA [24].

**Table 1:** Strains used.

Strain	Relevant genotype	Source or reference
BcATCC 14579	Reference wild-type *B.cereus* strain	[18]
Δ*plcRa*	Δ*plcRa*::*tet*	This study
Δ*abrB2*	Δ*abrB2*::*tet*	This study
Δ*plcRa-plcRa*	Δ*plcRa*::*tet*, pHT304Ω*plcRa ery*	This study
*plcRa’Z*	P*_plcRa_’-lacZ ery*	This study
*abrB2’Z*	P*_abrB2_’-lacZ ery*	This study
*yrrT’Z*	P*_yrrT_’-lacZ ery*	This study
*plcRa’Z-*Δ*plcRa*	P*_plcRa_’-lacZ ery* Δ*plcRa*::*tet*	This study
*abrB2’Z-*Δ*plcRa*	P*_abrB2_’-lacZ ery* Δ*plcRa*::*tet*	This study
*abrB2’Z-plcRa-*Δ*plcRa*	P*_abrB2_’-lacZ ery* pHT1618Ω*plcRa kana* Δ*plcRa*::*tet*	This study
*yrrT’Z-xyl’abrB2*	P*_yrrT_’-lacZ ery* pHT1618ΩP*_xyl_*’-*abrB2 kana*	This study
*yrrT’Z-xyl’abrB2-*Δ*plcRa*	P*_yrrT_’-lacZ ery* pHT1618ΩP*_xyl_*’-*abrB2 kana* Δ*plcRa*::*tet*	This study
*yrrT’Z-xyl’abrB2-*Δ*abrB2*	P*_yrrT_’-lacZ ery* pHT1618ΩP*_xyl_*’-*abrB2 kana* Δ*abrB2::tet*	This study
*abrB2’Z-xyl’papRa*	P*_abrB2_*’-lacZ *ery* pHT1618ΩP_xyl_’-*papRa kana*	This study
*abrB2’Z-xyl’papRa-*Δ*plcRa*	P*_abrB2_*’-lacZ *ery* pHT1618ΩP_xyl_’-*papRa kana* Δ*plcRa*::*tet*	This study

### Identification of PlcRa-controlled Genes

We characterized the regulatory role of PlcRa, by comparing the transcriptomes of the Δ*plcRa* and wild-type *B. cereus* ATCC 14579 strains during early stationary phase in LB medium. Assessments were carried out one hour after the onset of stationary phase (*t*_1_), when *plcRa* expression increases, and two hours after the onset of stationary phase (*t*_2_), when *plcRa* expression has reached a plateau.

In total, 117 genes were differentially expressed with a more than three-fold difference between the wild-type strain and the Δ*plcRa* mutant strain (Tables 2, 3). Forty-nine genes were more strongly expressed in the wild-type strain and 68 genes were less strongly expressed in the wild-type strain than in the mutant. In total, 12 genes were differentially expressed at *t*_1_, 88 were differentially expressed at *t*_2_ and 17 were differentially expressed at both *t*_1_ and *t*_2_. These transcriptome data were validated by RT-qPCR analysis on a set of 15 genes with new cultures (Tables 2, 3 and Table S1).

**Table 2:** Sulphur metabolism genes expressed more strongly in the wild-type strain than in the *B. cereus plcRa* mutant strain^a^.

		Wild type/Δ*plcRa* expression ratio		
		microarray analysis		qRT-PCR analysis
Locus tag^b^ *synonym* ^c^	Function/similarity	*t*_1_^d^	*t*_2_^d^	*t*_2_
BC1421 *cysH*	Phosphoadenosine phosphosulfate reductase	1	2	
BC1422 sat	Sulfate adenylyltransferase	1	6	21
BC1423 *cysC*	Adenylylsulfate kinase	1	8	
BC1424	Putative ferredoxin-nitrite reductase/sulfite reductase	1	5	32
BC1425	Hypothetical protein	1	5	
BC1426 *sumT*	Uroporphyrin-III C-methyltransferase	1	2	
BC1427 *sirB*	Sirohydrochlorin ferrochelatase	1	4	
BC1428 *sirC*	Precorrin-2 dehydrogenase	1	2	
BC4369 *yrrT*	AdoMet-dependent methyltransferase	1	12	8
BC4368 *mtnN*	methylthioadenosine/S-adenosylhomocysteine nucleosidase	1	4	
BC4367 *mccA*	Cystathionine ß-synthase	1	9	
BC4366 *mccB*	Cystathionine γ-lyase	1	16	15
BC4392 *yrvO*	Cysteine desulfhydrase	1	3	
BC4393 *cymR*	Cysteine metabolism repressor protein (RRF2 family)	1	3	
BC0075 *cysK*	OAS-thiol-lyase	1	3	
BC2617	Cysteine dioxygenase	1	2	
BC4003 *metE*	Cobalamin-independent methionine synthase	1	3	
BC4242 *tcyP*	Sodium-cystine symporter	1	7	3
BC4751	Putative sulfite reductase (flavoprotein alpha-subunit)	1	3	
BC4789 *luxS*	S-ribosylhomocysteine lyase/AI2 production	1	5	3

a. Genes with at least a three-fold difference in expression are shown. In the case of operons (probable or demonstrated) ratios below 3 were considered acceptable.

b. Locus tag in type strain ATCC 14579.

c. The gene names indicated correspond to *B. subtilis* homologs, with the exception of *dps2* (BA5290), which corresponds to a homologous gene in *B. anthracis*.

d. Cultures for RNA extraction were collected one hour (*t*_1_) or two hours (*t*_2_) after the entry into stationary phase.

**Table 3:** Genes expressed more strongly in the wild-type strain than in the *B. cereus plcRa* mutant strain^a^.

		Wild type/Δ*plcRa* expression ratio		
		microarray analysis		qRT-PCR analysis
Locus tag^b^ *synonym* ^c^	Function/similarity	*t*_1_^d^	*t*_2_^d^	*t*_2_
*Oxidative stress response*				
BC0518 *perR*	Peroxide stress response/metal-dependent repressor protein (Fur family)	3	1	
BC0377 *ahpC*	Alkyl hydroperoxide reductase small subunit	1	3	2
BC0376 *ahpF*	Alkyl hydroperoxide reductase large subunit	1	2	
BC5044 *dps2*	Dps-like miniferritin/antioxidant protein	3	2	3
BC4474 *ohrR*	Organic hydroperoxide resistance regulatory protein	1	3	
*Peptide/nickel transport*				
BC0242	Oligopeptide transport system permease protein oppB-like	1	4	3
BC0243	Oligopeptide transport system permease protein oppC-like	1	4	
BC0244	Oligopeptide transport ATP-binding protein oppD-like	1	5	3
BC0245	Oligopeptide transport ATP-binding protein oppF-like	1	3	
*Iron transport/metabolism*				
BC5380	Ferrichrome-binding protein	3	1	
BC5381	Ferrichrome transport ATP-binding protein fhuC	2	1	
BC5382	Ferrichrome transport system permease protein fhuG	3	1	
BC5383	Ferrichrome transport system permease protein fhuB	2	1	
BC1154	Ferrochelatase	4	1	
*Miscellaneous*				
BC2444	Putative transition state regulatory protein (AbrB) family) family)	6	3	50 (***t*** **_1_**)
BC3727 *yrhG*	Formate/nitrite transporter	3	1	
BC3225	Macrolide efflux protein	3	1	
BC4925	NADH dehydrogenase	3	1	
BC1224	Acetyltransferase	1	3	
BC3662 *ccdA*	Ribosomal-protein-alanine acetyltransferase	1	4	
BC3338	Hydrolase	1	3	
BC4660 *acuA*	Acetoin utilization protein AcuA	1	3	
BC1225	2'−5' RNA ligase	1	3	
*Hypothetical protein*				
BC2445	Hypothetical protein	3	2	
BC2446	Hypothetical membrane-spanning protein	2	2	
BC1074	Hypothetical protein	1	4	
BC3506	Hypothetical protein	1	4	
BC4208	Hypothetical protein	4	1	
BC5260	Hypothetical protein	1	3	
Total 29				

a,b,c,d. See Table 2 for legends.

Most of the proteins encoded by the genes upregulated by PlcRa (expressed more strongly in the wild-type strain) fell into four main categories: sulfur metabolism, oxidative stress responses, peptide transport and iron metabolism (Tables 2, 3). The largest category, sulfur metabolism, comprised 20 proteins, 18 of which were orthologs of proteins involved in cysteine transport and metabolism in *B. subtilis* (Table 2) (see below).

The second category, oxidative stress response proteins, contained five proteins, including PerR and OhR, two major regulators of the oxidative stress responses in *B. subtilis* [5] and presumably in *B. cereus* [25], [26]. PerR regulates many genes, including those of the *ahpCF* operon encoding the alkyl hydroperoxide reductase (AhpR), a detoxification system composed of two enzymes – hydrogen peroxide-forming NADH oxidase (nox-1) and peroxidase (AhpC) – that catalyzes the breakdown of molecular oxygen to hydrogen peroxide (H_2_O_2_) which is reduced by the second enzyme to water. This operon was downregulated in a *plcRa*–deficient mutant strain. The final protein in this group was a Dps-like miniferritin protein, the Dsp2 protein (BC5044), which is homologous to the Dsp2 protein of *B. anthracis* (BA5290) that has recently been shown to play a major role in oxidative stress resistance [27].

The third group comprises four components of an uncharacterized oligopeptide permease system, Opp, which is thought to be required for the import of small molecules into the bacterial cell. The fourth category consists of five proteins involved in iron transport and metabolism. In addition to these factors, PlcRa induces the expression of genes encoding proteins of various known or unknown functions. These genes include the gene displaying the strongest upregulation by PlcRa one hour after entry into stationary phase (Table 3). This gene (BC2444) encodes a regulatory protein belonging to the AbrB family analyzed in greater detail below.

Most of the proteins encoded by genes downregulated by PlcRa (lower expression in the wild-type strain versus *plcRa*-deficient mutant strain) belonged to two major categories (Table S1). The first one consisted of general stress Sigma factor SigB, its associated regulatory proteins RbsV, RbsW and RbsP and six SigB-controlled proteins [28]. The second consisted of 31 prophage proteins encoded by the genes of two prophages, *phBC6A52* and *phBC6A51*, harbored by the chromosome of strain ATCC 14579 [18]. Our transcriptome analysis indicated that PlcRa downregulated the expression of about 50% of *phBC6A52* genes (49, total ORFs number) and 13% of *phBC6A51* genes (75, total ORFs number) [29]. BC1852 and BC1857 encode *phBC6A51* prophage proteins thought to be involved in DNA repair: an SbcC-like chromosomal ATPase and an SbcD-like protein, both related to bacterial SMC-like (structural maintenance of chromosome) proteins [30]. This is the first report of a bacterial regulator controlling the expression of numerous phage genes in this strain, or even in *B. cereus* [29], [31], [32]. PlcRa downregulated several other genes unrelated to these two main categories, including a three-gene operon encoding the components of the Hbl enterotoxin, a virulence determinant thought to be involved in diarrheal disease. Expression of the *hbl* operon is activated by PlcR at the onset of the stationary phase [1]. PlcRa is a pleiotropic regulator activated at the onset of stationary phase. Since PlcRa controls regulators, some PlcRa-controlled genes may be indirect PlcRa targets.

### PlcRa Upregulates Cysteine Metabolism Genes

Twenty of the 49 genes upregulated by PlcRa encode proteins involved in sulfur metabolism (Table 2). These genes were found to be differentially expressed only at *t*_2_. The sulfur metabolism genes of *B. cereus* remain poorly annotated, despite the availability of several *B. cereus* genomes. We therefore reconstructed the sulfur metabolism pathway, by searching for orthologs of *B. subtilis* genes in the *B. cereus* ATCC 14579 genome. The transport of sulfur sources [33] and the two major cysteine biosynthetic pathways in *B. subtilis –* the thiolation pathway, which requires sulfide, and the reverse transsulfuration pathway, which converts homocysteine to cysteine, with cystathionine formed as an intermediate [34], [35] *–* are conserved in *B. cereus* ATCC 14579. We were able to identify all the enzymes and transporters required for these pathways other than those for the reduction of sulfite to sulfide (Table 2, Figure S1).

Eighteen PlcRa-controlled genes were identified as putative homologs of genes involved in cysteine metabolism in *B. subtilis* (Table 2, Figure S1): a cystine (the oxidized form of cysteine) transporter (*tcyP*) [33] and proteins involved in the biosynthesis of cysteine from sulfate (the *cysH* operon and *cysK* gene) or methionine (the *yrrT-mtnN-mccAB* operon and *luxS* gene) [35]. In *B. subtilis*, the expression of these genes is repressed by the transcriptional regulator CymR, in response to cysteine availability [35], [36]. BC4393, which is 76% identical to CymR from *B. subtilis*, is probably the global negative regulator of cysteine metabolism in *B. cereus*. By screening for the *B. subtilis* CymR box sequence, we identified putative CymR-binding motifs upstream from *cysK*, *cysH*, *tcyP*, *yrrT* and *luxS* (Figure S2). Thus, PlcRa upregulates 14 probable members of the CymR regulon in *B. cereus.* The *cymR* gene itself was downregulated in a *plcRa* mutant strain (ratio 3, Table 2). A *lacZ* transcriptional fusion was constructed with the promoter region of the *cymR* gene and introduced into the wild-type strain and the *plcRa* mutant. We found that the *cymR* gene expression was constitutive (Figure S3) as described in *B. subtilis* (I. Martin-Verstraete, unpublished results, [37]). A significant but small difference (ratio 2) in β-galactosidase activity was transiently observed between the wild-type strain and the *plcRa* mutant over a short period, one hour after the onset of stationary phase (Figure S3). We hypothesized that PlcRa activated CymR-controlled genes independently of CymR or that PlcRa upregulated CymR-controlled genes through the modulation of CymR activity.

### Transcriptional Control of the BC2444 (*abrB2*) Gene by PlcRa

**Figure 4:**
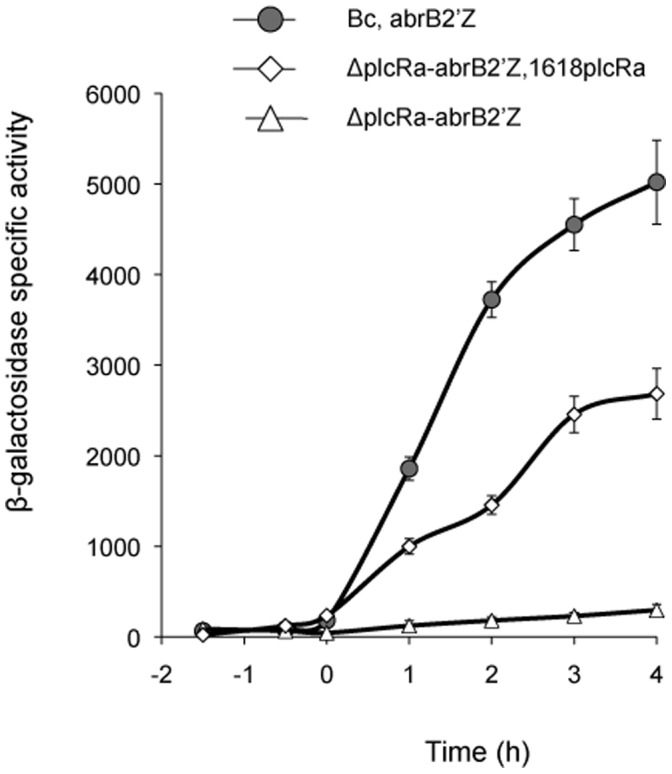
PlcRa activates *abrB2* gene expression early in stationary phase. β-galactosidase specific activity (U/mg protein) of the wild-type (black circles), Δ*plcRa* (white triangles) and complemented Δ*plcRa* (white diamonds) strains harboring the transcriptional P*_abrB2_*’-*lacZ* fusion, in LB. Errors bars are shown.

For characterization of PlcRa as a transcriptional regulator, we searched for a direct target gene candidate. Microarray analysis indicated that the PlcRa-regulated gene BC2444 was differentially expressed at both *t*_1_ and *t*_2_ and that this gene presented the highest differential expression ratio at *t*_1_ (6-fold in the microarray analysis and 50-fold in RT-qPCR analysis) (Table 3). This gene encodes a putative regulator 50% identical to the AbrB regulator of *B. subtilis* [38] and 85% identical to that of *B. cereus* (BC0042) [39]. We therefore renamed BC2444, *abrB2*. Given the high levels of early activation of this regulator gene observed here, we decided to investigate its expression kinetics. We analyzed the expression of a plasmid-borne P*_abrB2_’-lacZ* transcriptional fusion in the wild-type and Δ*plcRa* strains (Figure 4). We found that *abrB2* expression increased sharply from *t*_0_ to *t*_2_. In the *plcRa* mutant, *abrB2* expression was strongly reduced while the introduction of *plcRa* in trans restored its expression. Thus, *abrB2* expression is activated by the presence of PlcRa at the onset of stationary phase.

### Purified PlcRa Binds Specifically to the *abrB2* Promoter in the Presence of PapRa_7_

**Figure 5:**
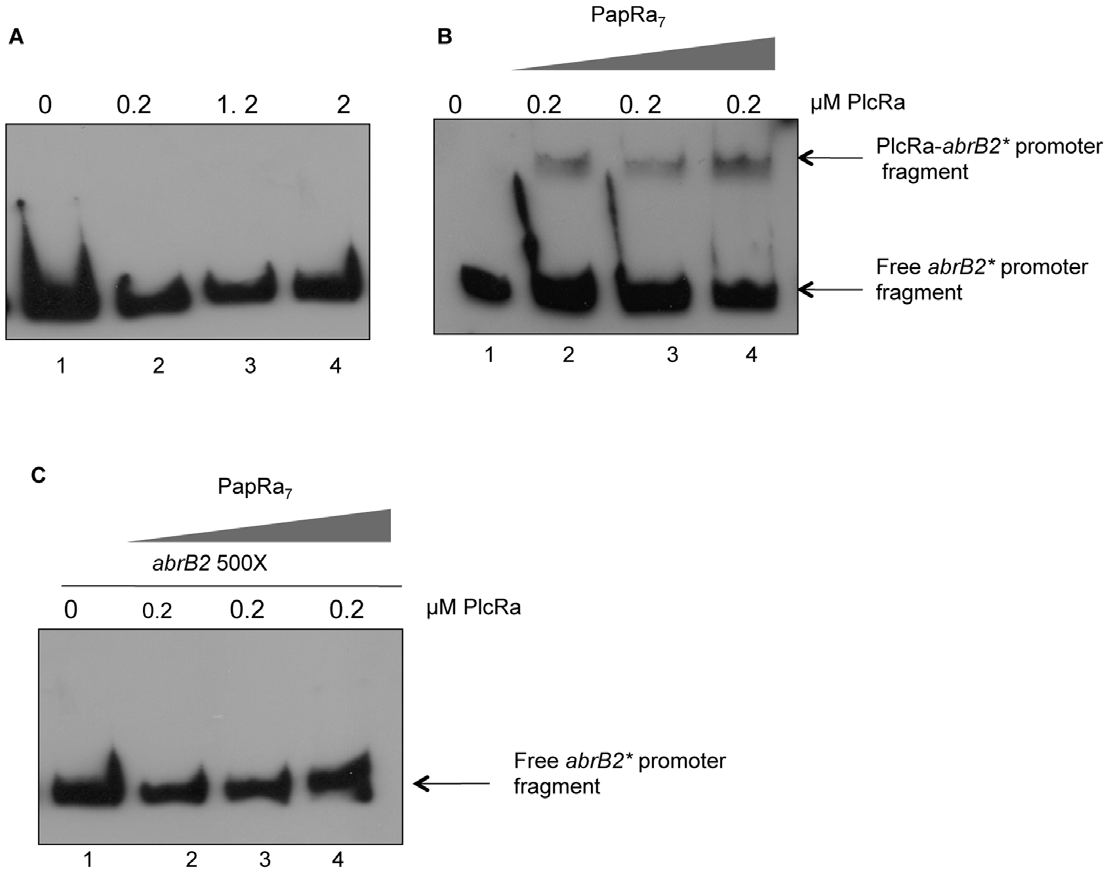
Electrophoretic mobility shift assay to determine conditions of PlcRa binding to *abrB2* promoter region. Fragment was generated by PCR amplification and end labeled with biotine. A constant amount of probe (5 fmol) was incubated at room temperature with the indicated concentrations of PlcRa without (A) or with PapRa_7_ (B, C) at these concentrations: 0.2 µM (well 1, 2), 2 µM (well 3) and 20 µM (well 4) final concentration. C. The EMSA was carried out in the presence of 500-fold excess (wells 1–4) of the same unlabeled PCR-amplified DNA. Samples were run on 6% non-denaturing polyacrilamide gels.

To further understand the role of PlcRa on the *abrB2* expression and distinguish between direct or indirect effects, electrophoretic mobility shift assays (EMSA) were performed with the same fragment present in plasmid P*_abrB2_’*-lacZ (Figure 4) using purified PlcRa. A biotin end-labeled DNA fragment containing the *abrB2* promoter region was incubated in the presence of increasing PlcRa concentrations (Figure 5A). We did not observe gel-retardation under these conditions.

All characterized RNPP regulators are activated through a secreted signaling peptide that interacts with the TPR activation domain. As described above, the PapRa_7_ heptapeptide (CSIPYEY) is a relevant candidate to act as a signaling peptide for PlcRa activation. Synthetic PapRa_7_ at different increasing concentrations was incubated with PlcRa without modifying the binding buffer (Figure 5B). A protein-DNA complex was formed under these conditions. In order to confirm that PlcRa binding to the *abrB2* promoter region was specific, the EMSA was carried out in the presence of PapRa_7_ and a 500-fold excess of the same unlabeled PCR-amplified DNA (Figure 5C). As shown in Figure 5B, the shift observed when we incubated the *abrB2* promoter DNA fragment with PlcRa in the presence of PapRa_7_, disappeared in the presence of an excess of the same fragment. An additional EMSA control was carried out in the presence of PapRa_7_ and a new biotin end-labeled DNA fragment containing the *ilsA* promoter region [1]. We did not observe gel-retardation under these conditions (data not shown). Our results indicated that PlcRa is the direct regulator of *abrB2* and that its binding to the *abrB2* promoter region *in vitro* requires the presence of PapRa_7_.

### The Addition of PapRa_7_ Enhanced *abrB2* Expression *in vivo* in a PlcRa-dependent Manner

**Figure 6:**
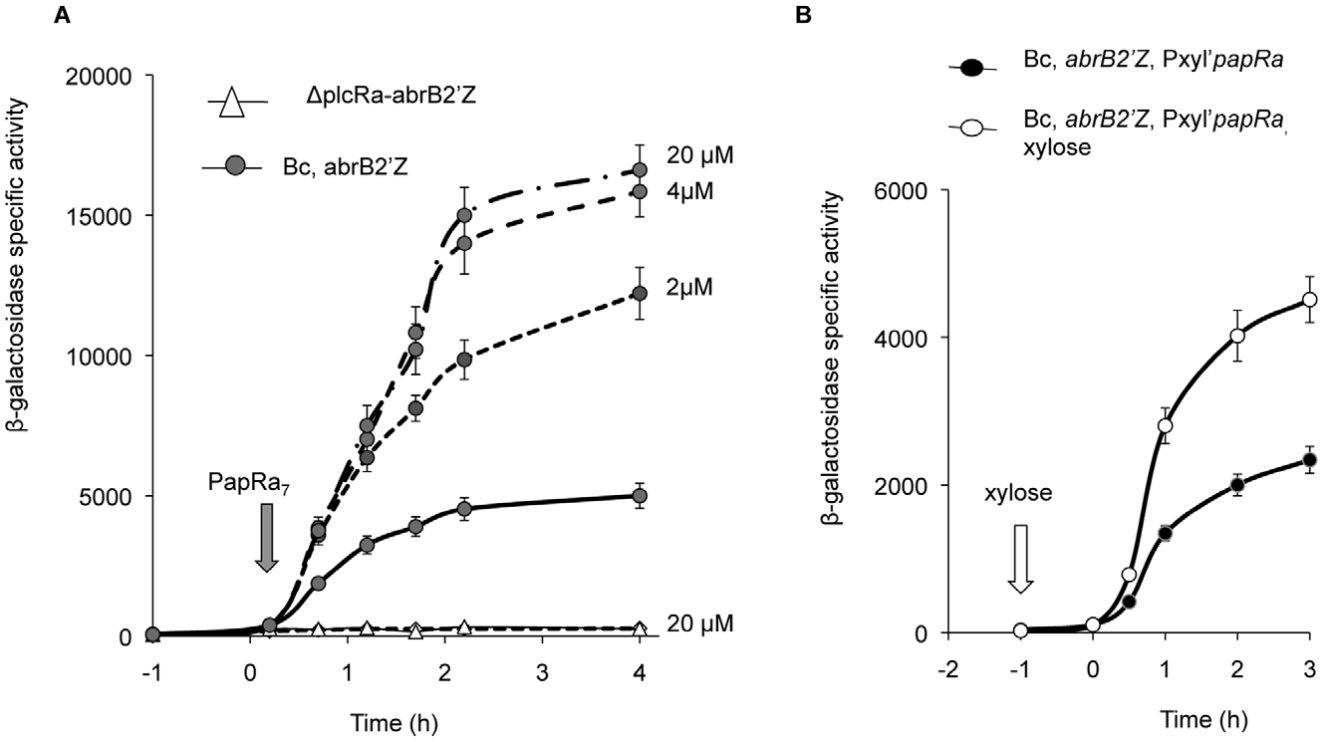
Addition of synthetic PapRa_7_ or overexpression of *papRa* enhanced *abrB2* gene expression in a PlcRa-dependent manner. A. Expression of the P*_abrB2_*’-*lacZ* fusion in the wild-type and in the Δ*plcRa* mutant strains in the presence of synthetic PapRa_7_. The cells were grown at 37°C in LB medium and PapRa_7_ was added at *t*_0.2_ (onset of stationary phase) at different concentrations: 2 µM or 4 µM or 20 µM. Dashed lines correspond to LB cultures with PapRa_7,_ and thick line corresponds to LB culture without PapRa_7_. B. Expression of the P*_abrB2_*’-*lacZ* transcriptional fusion in the wild-type strain carrying pHT1618P_xyl_’-*papRa*. The cells were grown at 37°C in HCT medium in the presence or absence of 10 mM xylose. Xylose was added at *t*_−1_ as indicated by a white arrow.

We then monitored the expression of the P*_abrB2_*’-*lacZ* fusion in the wild-type strain, after the addition of synthetic PapRa_7_ at various concentrations at the start of the stationary phase (Figure 6A). β-galactosidase activity increased by a factor of two to three after the addition of PapRa_7_ at concentrations of at least 2 µM in wild-type strain cultures whereas the addition of this peptide to cultures of a Δ*plcRa* mutant had no effect (Figure 6A). We observed no increase in β-galactosidase activity when PapRa_7_ was added to the culture after t_2.5_. Moreover, when the peptide was added to the culture during the exponential growth phase, the increase in β-galactosidase activity coincided strictly with entry into stationary phase (data not shown). The addition of synthetic PapRa_7_ in the culture positively affected *abrB2* expression at the onset of stationary phase in a PlcRa-dependent manner. A plasmid harboring the *papRa* gene under the control of a xylose-inducible promoter was constructed and subsequently introduced into a wild-type strain containing the P*_abrB2_*’-*lacZ* and into the *plcRa*-deficient mutant previously described. When xylose was added at *t*_−1_, the β-galactosidase activity strictly increased at the onset of stationary phase compared to the level observed in the culture without xylose (Figure 6B) whereas in the *plcRa* mutant we did not observe any change of the weak β-galactosidase activities (data not shown). We demonstrated that the level of PapRa production influenced *in vivo* the activity of PlcRa.

### AbrB2 Controls *yrrT* Expression

**Figure 7:**
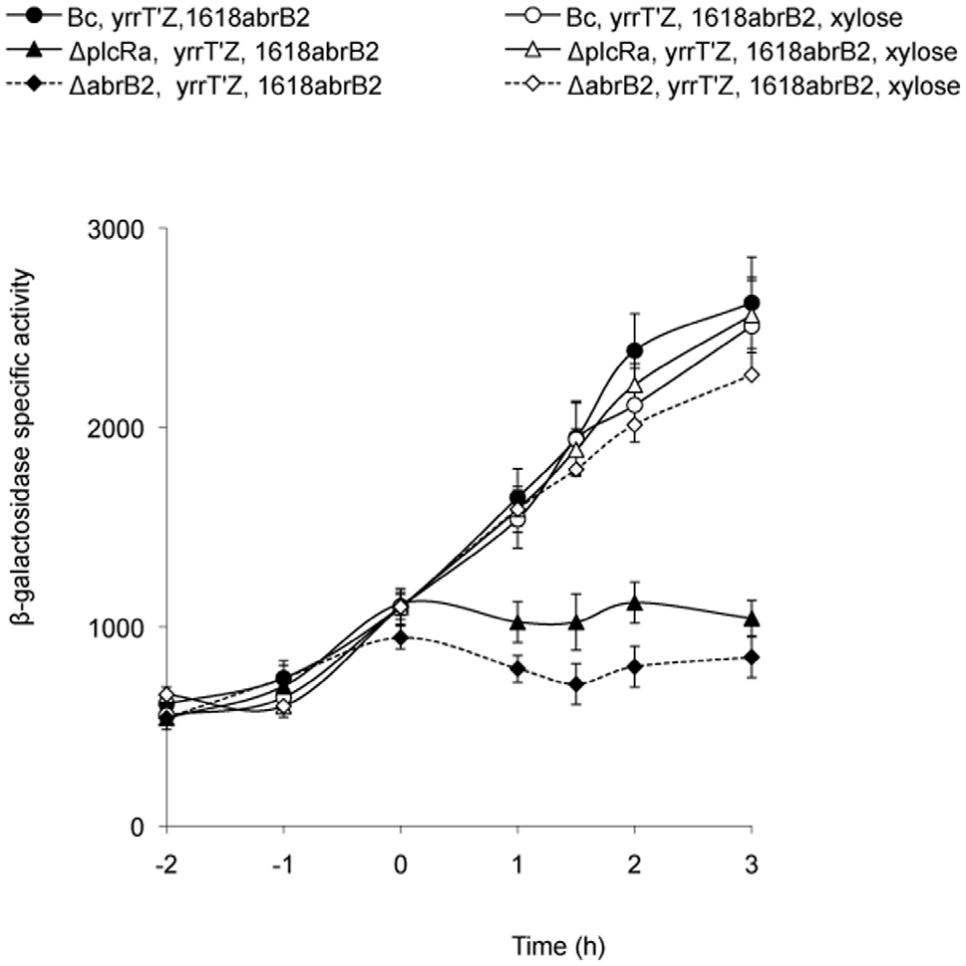
AbrB2 controls the expression of *yrrT* operon, involved in methionine to cysteine conversion. β-galactosidase specific activity (U/mg protein) of wild-type (black circles), Δ*plcRa* (black triangle) and Δ*abrB2* (black diamonds) strains harboring both pHT304*_yrrT_’-lacZ* and pHT1618KΩP*_xyl_*-*abrB2* plasmids, in HCT. See legends figure 5 for growth conditions. White symbols indicate cultures in the presence of xylose_._

To investigate the possible role of AbrB2 in the regulation of PlcRa-controlled genes, we constructed a deletion mutant strain (Table 1). We tested the effect of *abrB2* deletion on the transcription of the *yrrT* operon, which encodes proteins involved in methionine-to-cysteine conversion [34] (Figure S1). We constructed a transcriptional fusion between *lacZ* and the *yrrT* promoter region and investigated the kinetics of P*_yrrT_'-lacZ* expression in the wild-type and Δ*plcRa* and Δ*abrB2* mutant strains during growth. To understand the role of AbrB2 on *yrrT* expression, we expressed *abrB2* under the control of a xylose-inducible promoter (P*_xylA_*) in pHT1618. We introduced this plasmid into the wild type, Δ*plcRa* and Δ*abrB2* strains containing the P*_yrrT_'-lacZ* fusion (Figure 7). In the absence of xylose, an increase in *β*-galactosidase activity was detected at the onset of stationary phase in the wild-type strain, but not in either of the mutant strains (Figure 7). In the presence of xylose, *β*-galactosidase activity increased at the onset of stationary phase in both the *plcRa* and *abrB2* mutant strains, reaching levels similar to those for the wild type. Thus, the down-regulation of *yrrT* expression due to *abrB2* inactivation was complemented by *abrB2* in trans. In addition, the expression of *abrB2* under the control of the *xylA* promoter in the presence of xylose also restored the expression of the *yrrT* fusion in a Δ*plcRa* background. These findings demonstrated that the PlcRa-dependent control of *yrrT* is mediated by AbrB2.

### High Sensitivity of the *plcRa* Mutant Strain to Peroxide and Disulfide Stresses

**Figure 8:**
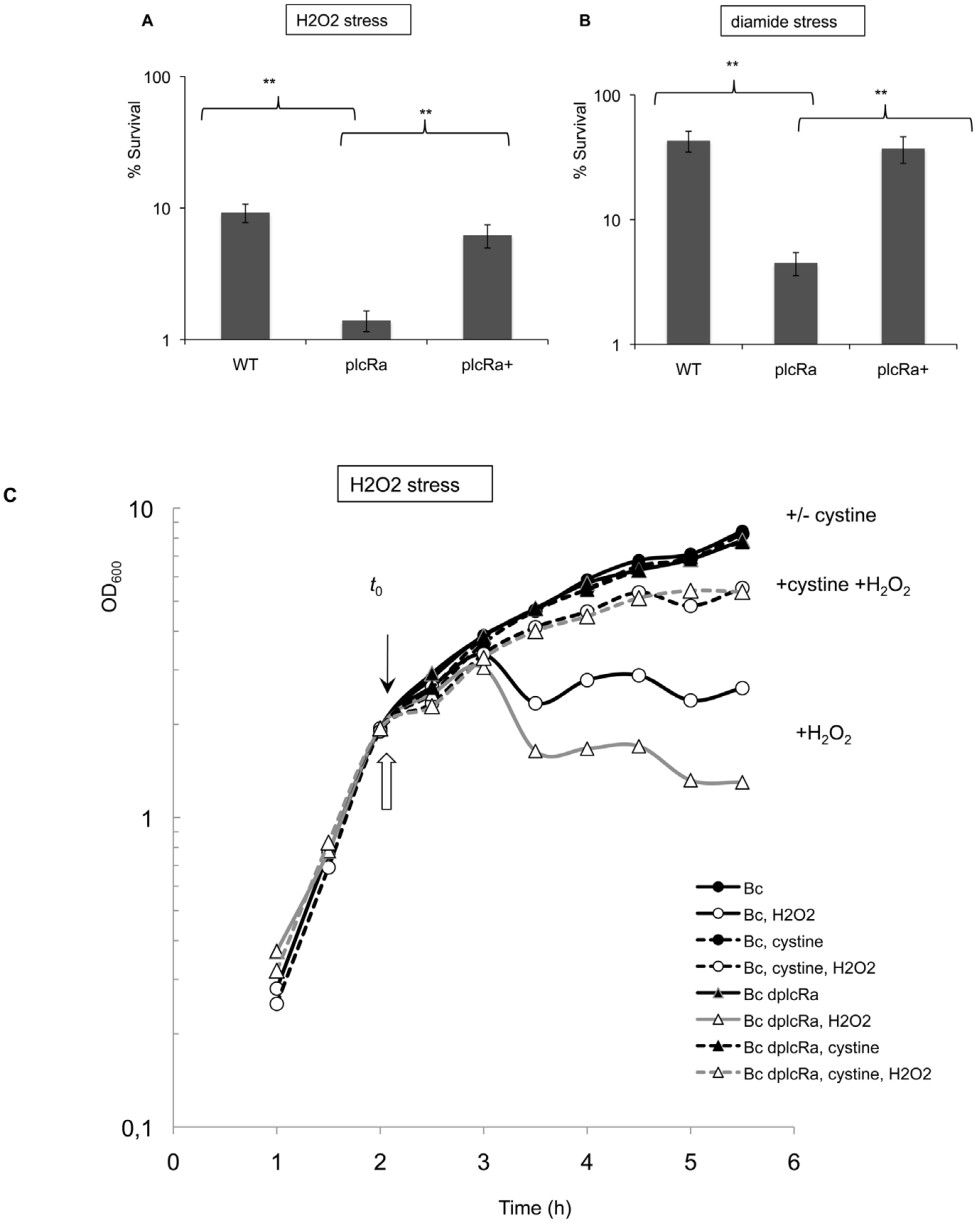
Sensitivity to peroxide and disulfide stress of a *B. cereus plcRa* mutant. We assessed the viability of wild-type (WT), Δ*plcRa* (plcRa) and complemented Δ*plcRa* (plcRa+) strains in early stationary phase. Early stationary-phase cells grown in LB medium (∼ OD 3, ∼ *t*_0.4_) were treated for 10 minutes with 1 mM H_2_O_2_ (A) or for 40 minutes with 10 mM diamide (B) in LB and plated on LB. The results shown are the mean values for survival, expressed as a %, with standard deviations, and are representative of three independent experiments.**: *P*<0.01. C. The addition of cystine strongly improved the peroxide stress resistance of the *plcRa* mutant. We assessed the growth inhibition of wild-type and Δ*plcRa* strains in early stationary phase. Growth curves of the wild-type strain (black circles) and the mutant (black triangles) in LB medium without (solid line) or with 1 mM cystine (dashed line). Hydrogen peroxide (0.4 mM) was added at an OD of 2 (∼ *t*_−0.3_). White symbols indicate cultures treated with H_2_O_2_. *t*_0_ is indicated by a black arrow and hydrogen peroxide addition by a white arrow. This experiment was carried out four times and the results of one representative experiment are shown.

PlcRa controls the expression of genes encoding proteins involved in the oxidative stress response and cysteine biosynthesis. Previous studies in *B. subtilis* and *S. aureus* have established strong links between cysteine metabolism and oxidative stress [5], [35], [37], [40]. It has been shown in *B. subtilis* [5] and *B. anthracis* [41], [42], [43] that cysteine itself and cysteine-containing molecules such as bacillithiol or CoenzymeA play a key role in protection against oxidative stress. We therefore evaluated the sensitivity of the wild-type and *plcRa* mutant strains to H_2_O_2_ and diamide, a compound that causes thiol oxidation and disulfide stresses. We first demonstrated that the addition of H_2_O_2_ (1 mM) or diamide (10 mM) to LB medium at the start of stationary phase had no dramatic effect on *plcRa* expression (Figure S4). The viability of the Δ*plcRa* and wild-type strains was then assessed after the addition of H_2_O_2_ (1 mM) or diamide (10 mM) to the LB medium (Figure 8AB). Survival rates for the Δ*plcRa* strain were lower than those for the wild-type strain, by a factor of six in the presence of H_2_O_2_ and 10 in the presence of diamide. The introduction of *plcRa,* in *trans*, into the Δ*plcRa* strain restored the wild-type phenotype. Thus, *plcRa* inactivation led to an increase in sensitivity to H_2_O_2_− and disulfide-induced stresses, suggesting a role for PlcRa in the regulation of the peroxide and disulfide stress defense system of *B. cereus*.

### The Addition of Cystine Improves the Stress Resistance of the *plcRa* Mutant

In *B. subtilis*, cysteine depletion induces the expression of cysteine synthesis genes, such as those of the *cysH* operon, which is involved in cysteine production from sulfate, or the *yrrT* operon, which is involved in methionine-to-cysteine conversion [34], [44]. These operons were downregulated in the *plcRa* mutant (Table 2, Figure S1). We hypothesized that this would result in lower intracellular levels of cysteine in the *plcRa* mutant. Moreover, we found that the expression of the P*_yrrT_*'-*lacZ* fusion was induced at the onset of stationary phase in the wild-type strain, suggesting cysteine depletion in the growth medium leading to the induction of the methionine-to-cysteine conversion pathway. No such induction was observed in the *plcRa* mutant strain (Figure 7). We thus investigated the effects of cystine addition during peroxide stress. Cystine (1 mM) was added to the culture in mid-exponential growth phase. We first demonstrated that the addition of cystine to LB medium in mid-exponential growth phase did not modify *plcRa* expression (data not shown). No growth difference was observed for the wild-type and Δ*plcRa* mutant strains with or without cystine (Figure 8C). Two hours later, at the end of exponential phase, H_2_O_2_ (0.4 mM) was added to the medium. Both strains presented a growth arrest one hour after the H_2_O_2_ addition which was characterized by a OD_600_ measurements drop. In addition, in the presence of H_2_O_2,_ OD_600_ measures of the Δ*plcRa* mutant strain were lower than the wild type strain. When cystine was added, the growth arrest for both strains was abolished (Figure 8C). Thus, cystine significantly reduced the sensitivity of these cells to H_2_O_2_ stress in our growth conditions. Moreover, these results strongly suggest that cystine transport is efficient in the Δ*plcRa* mutant, as in the wild type and it might be due at least partly to the TcyABC system (BC0872–BC0873–BC0874) that is not controlled by PlcRa (Figure S1). These results demonstrated a role for PlcRa in the regulation of the oxidative stress defense system of *B. cereus* in relation with cysteine biosynthesis.

## Discussion

We characterized PlcRa, a new member of the RNPP family of transcriptional regulators in the *B. cereus* group. All RNPP regulators are activated through a secreted signaling peptide that interacts with the TPR activation domain [12]. Our comparative modeling of the PlcRa protein indicates a folding similar to PlcR, with a DNA-binding domain and five TPR motifs putatively involved in the peptide binding. A small gene, *papRa,* encoding a putative exported peptide is present upstream from *plcRa*. Based on a sequence alignment with the PapR peptide, the CSIPYEY fragment -PapRa_7_− was proposed as a good candidate for a signaling heptapeptide. PapRa_7_ corresponds to an internal region of the carboxy-terminal part of PapRa. This is dissimilar to findings for PapR whose mature form corresponds to the C-terminal end [12]. We first demonstrated that in the *plcRa* mutant, *abrB2* expression was strongly reduced while the introduction of *plcRa* in trans restored its expression. Then, we demonstrated *in vitro* that PlcRa binds specifically to the *abrB2* promoter, and that its binding requires the presence of PapRa_7_. Moreover, the addition of this heptapeptide in the culture as well as the overexpression of the *papRa* gene enhanced *abrB2* expression significantly, in a PlcRa-dependent manner, indicating that PapRa modulates PlcRa activity. Taken together, our data suggest that PapRa, in the form of PapRa_7,_ can function as an extracellular signal. In addition, the expression of *plcRa* and *abrB2* genes was strongly activated at the onset of stationary phase suggesting a transcriptional regulation in relation with cell density. Together, our data indicate that PlcRa/PapRa is probably a new quorum sensing system in *B. cereus*. The production and the maturation of PapRa and the binding of PapRa_7_ with the TPR activation domain of PlcRa remain to be established.

The promoter region of PlcR-regulated genes contains a highly conserved palindromic sequence (TATGNAN4TNCATA), constituting the PlcR binding site [7]. Despite the structural similarities between PlcR and PlcRa, no palindromic sequence was found in the promoter regions of PlcRa-regulated genes, including *abrB2*, a direct PlcRa target. We were unable to identify a conserved motif upstream from *abrB2* and other PlcRa-controlled genes, using various bioinformatic tools.

The timing of *plcRa* expression (*t*_0.5_ to *t*_1.5_) suggests an additional regulatory mechanism that prevents constitutive expression by SigA and determines stationary phase expression. We have shown that the *plcRa* gene is not autoregulated. In light of knowledge of the regulatory network controlling transition state in *B. subtilis* model [3] we can speculate for a switch from vegetative sigma to transition sigma factor or for the activation of an activator or for the inactivation of a repressor. Overall these results suggest a tightly controlled *plcRa* expression at the onset of stationary phase, and this regulatory mechanism, different from the *plcR* expression activation [7], [8], remains to be elucidated.

PlcRa principally positively regulates the transcription of genes involved in regulation, cysteine synthesis and oxidative stress resistance. It also downregulates the expression of numerous phage genes and this regulation may be indirect or direct. We investigated the expression and the role of a major PlcRa directed gene, *abrB2*. This gene encodes an AbrB-like regulator, and displays the highest level of upregulation at *t*_1_ whereas most of the genes (35/49) positively controlled by PlcRa displays upregulation only at *t*_2_. This could suggest that PlcRa may regulate gene expression indirectly, via AbrB2 at least for the genes induced at *t*_2_. This is the case for the *yrrT* operon encoding proteins involved in methionine-to-cysteine conversion: the expression of *abrB2* under the control of a xylose-inducible promoter bypasses the requirement for PlcRa for *yrrT* expression.

Our results indicate the existence of links between PlcRa and the response(s) to stress stimuli. Indeed, we showed that the Δ*plcRa* mutant is more sensitive to H_2_O_2_ and diamide stresses than the isogenic wild-type strain. *B. cereus* group species respond to oxidative stress by the activation of different cellular defence mechanisms. These are composed of scavenging enzymes, as well as protection and repair systems presumably organized in highly sophisticated networks [5], [25], [26], [45]. Our transcriptome analysis showed the downregulation of one iron uptake system in the *plcRa-*deficient mutant (Table 3). An induction of iron and manganese uptake systems in response to H_2_O_2_ stress has been demonstrated in a *B. cereus* ATCC 14579 transcriptome study [25], and in *B. anthracis* combined proteomic and transcriptomic analysis [45]. H_2_O_2_ stress induces the synthesis of many proteins and enzymes, such as catalases, thioredoxin reductase, ferroxidase and peroxidases, responsible for eliminating H_2_O_2_ from the cells [25], [45], [46]. We therefore suggest that the lower level of resistance to H_2_O_2_ stress in the *plcRa* deficient mutant may be at least partially due to the lower expression level of AhpCF, a major two-enzyme detoxification system, and/or Dps2, a Dps-like miniferritin (Table 3) [27]. One of our results was apparently contradictory: *perR* (ratio 3, Table 3) which encodes a repressor, was found to be weakly expressed in a *plcRa* mutant strain, together with PerR*-*presumably controlled genes, *ahpCF* operon and *dps2* gene. It was also observed in *B.cereus* [25] and in *B.anthracis* [45] that H_2_O_2_ treatment modified the expression of both PerR-controlled genes and the *perR* gene itself in the same manner, rather than in the opposite manner as expected. However, this increased RNA level was not correlated with an increased protein level [45] and it is well established in *B. subtilis* that PerR is activated through conformational change [5].

In the Δ*plcRa* mutant, the expression of genes encoding proteins involved in cysteine synthesis from sulfate or methionine is downregulated (Table 2, Figure S1), and cystine addition to a Δ*plcRa* culture improved H_2_O_2_ stress resistance (Figure 8C). It has previously been shown that modifications in the intracellular concentration of cysteine lead to increased sensitivity to oxidative stresses [5], [37], [40], [47]. For example, in *B. subtilis* and *Staphylococcus aureus*, *cymR*-deficient strains, which accumulate cysteine due to the derepression of genes involved in cysteine synthesis, are highly sensitive to H_2_O_2_, disulfide, paraquat, copper- and tellurite-induced stresses [40], [47]. Indeed, the range of acceptable intracellular cysteine concentrations is narrow, as this concentration must be kept below the toxicity threshold but above the minimum requirement for protein synthesis and the production of essential molecules, including compounds required for thiol homeostasis, which plays an important role in protection against oxidative stress [5], [41]. We suggest that the *plcRa* mutant had a lower intracellular cysteine concentration, resulting in a higher susceptibility to both H_2_O_2_ and disulfide stresses generated by the thiol oxidant diamide. Indeed, cysteine is the direct precursor of low-molecular weight (LMW) thiol molecules such as bacillithiol [41] and Coenzyme A [42], [43]. These molecules are the key actors in the maintenance of cytosolic redox balance and in adaptation to the presence of reactive oxygen species in the bacteria of the *B. cereus* group [41], [42], [43]. In addition, S-thiolation by cysteine, the most abundant LMW thiol in cells, constitutes a general mechanism of thiol protection of proteins in *B. subtilis* [48] after oxidative stress, and remains to be characterized in *B. cereus* group.

The regulation by PlcRa of one of the key operons involved in cysteine synthesis from methionine is mediated by AbrB2. We suggest that AbrB2 regulates the expression of genes encoding proteins involved in cysteine metabolism**.** These genes probably belong to the CymR regulon. Further investigations are required to determine the molecular mechanisms by which AbrB2 regulates the set of genes encoding proteins involved in cysteine metabolism which may be direct or may involve CymR control.

Overall, our results demonstrate for the first time the existence of regulatory connections between cysteine metabolism and the oxidative stress responses at the onset of stationary phase in the *B. cereus* ATCC 14579 strain. These connections are partly controlled via the PlcRa and AbrB2 regulators, which are found exclusively in the *B. cereus* group. We have shown that PlcRa is connected to regulons which are probably involved either directly (OhR, PerR) or indirectly (CymR) in the responses to peroxide and disulfide stresses in *B. cereus*. Finally, *plcRa* inactivation had no significant effect on sporulation capacity in many common laboratory conditions (data not shown). We therefore suggest that the involvement of PlcRa in stress responses may ensure bacterial survival during the transition state preceding the initiation of sporulation. In conclusion, PlcRa is a new pleiotropic RNPP regulator, involved in major physiological processes in bacteria of the species *B. cereus*: adaptation to poor sulfur source conditions and oxidative environment early in stationary phase.

## Materials and Methods

### Bacterial Strains and Growth Conditions

The type strain *B. cereus* ATCC 14579 [18] was used throughout this study. *Escherichia coli* K-12 strain TG1 was used as a host for cloning experiments [49]. Plasmid DNA for the electroporation of *B. cereus* was prepared from *E. coli* strain ET12567 (Stratagene, La Jolla, CA, USA) [50]. *E. coli* and *B. cereus* cells were transformed by electroporation, as previously described. *E. coli* and *B. cereus* strains were grown at 37°C in Luria Bertani (LB) broth and, where indicated 1 mM cystine was added to the culture medium for *B. cereus*. The following antibiotic concentrations were used for bacterial selection: ampicillin, 200 µg ml^−1^ for *E. coli*; kanamycin, 200 µg ml^−1^; tetracycline, 15 µg ml^−1^ and erythromycin, 5–10 µg ml^−1^ for *B. cereus*. Bacteria producing β-galactosidase were identified on LB plates containing X-Gal (40 mg ml^−1^). The *xylA* promoter was induced in *B. cereus* by adding xylose (10 mM, final concentration) to the HCT 0.2% glucose culture medium. For microarray analysis, qPCR and β-galactosidase assays, all *B. cereus* strains were grown in LB broth at 37°C, in flasks, with an aeration ratio of 10, on a rotary shaker operating at 175 rpm. Cultures were inoculated, to an OD_600_ of 0.05, with cells in the exponential growth phase in LB broth, and culture pellets were harvested between 2 h before (*t*_−2_) and 4 h after (*t*_4_) the onset of stationary phase, for β-galactosidase assays. Cells were harvested at *t*_1_ and *t*_2_ for DNA microarray analysis and for qPCR analysis for DNA microarray validation and at *t*_0,5_, *t*_1,5_ and *t*_2,5_ for qPCR expression analysis. The onset of the stationary growth phase (*t*_0_) was defined as the breakpoint in the slope of the exponential phase growth curve [8].

### DNA Manipulation Techniques

Chromosomal DNA was extracted from *B. cereus* cells with the Puregene DNA Purification kit (Gentra Systems, USA). Plasmid DNA was extracted from *E. coli* on QIAprep spin columns (QIAGEN, France). Restriction enzymes (New England Biolabs, USA) and T4 DNA ligase (New England Biolabs, USA) were used in accordance with the manufacturer’s recommendations. Oligonucleotide primers for molecular constructs were synthesized by Sigma-Proligo (Paris, France) and primers for qPCR were synthesized by Eurofins-MWG (Paris, France). PCR was performed in an Applied Biosystems 2720 Thermak cycler (Applied Biosystem, USA). Amplified fragments were purified with the QIAquick PCR Purification Kit (QIAGEN, France). Digested DNA fragments were extracted from gels with the QIAquick Gel Extraction Kit (QIAGEN, France). Nucleotide sequences were determined by Cogenics (Meylan, France).

### Rapid Amplification of 5′-cDNA Ends (5′RACE)

A 5′RACE experiment was conducted to map the transcriptional start site of *plcRa*. *B. cereus* cultures were grown for four hours (*t*_1_). RNA (4 µg) was used for cDNA synthesis with the Superscript IITM reverse transcriptase (Invitrogen) and a gene-specific primer (BC0988GSP1: 5′-TCAGAATTAGTTAAAGTCAGTT-3′). The resulting cDNA was purified on S.N.A.P columns and a poly(dC) tail was added (Invitrogen 5′RACE system). The dC-tailed cDNA was amplified by PCR with an Abridged Anchor primer (Invitrogen) and a second gene-specific primer (BC0988GSP2: 5′-GCAATTGCTTCATGCCACATTAGA-3′), complementary to a region upstream from the binding site of the GSP1 primer. PCR products were isolated by gel extraction and inserted into the pGEM-T easy cloning vector (Promega). Three independent PCR products were sequenced.

### Construction of Deletion Strains

The chromosomal *B. cereus plcRa* and *abrB2* genes were disrupted by homologous recombination with the pRN5101 heat-sensitive vector [51]. For the *B. cereus plcRa* and *abrB2* mutant constructs, a tetracycline cassette carrying the *tet* gene was used for cloning [52] (Table 1). Each molecular construct, containing the 5′- and 3′- end flanking regions of the target gene and the positive selection cassette, was inserted between the *Hin*dIII and *Bam*HI sites of pRN5101.

*Bam*HI-*Pst*I and *Xba*I-*Hin*dIII DNA fragments corresponding to the regions upstream and downstream from the *plcRa* gene were amplified from the *B. cereus* chromosome by PCR, with the primers R1–1 (5′-GC**GGATCC**ATGTTGAACATGTTTTAAATAC-3′) R1–2 (5′-AA**CTGCAG**TTTTTCAATCTTGCTAATTTG-3′) and R1–3 (5′-GC**TCTAGA**TGTTTATTAAAAATGAAACAAC-3′) R1–4 (5′-CCC**AAGCTT**TGAAAGAAGTTTAGGATATTC-3′). External primers R1–V1 (5′-GATCAAATCGCAAAAAGGCACCTTAG-3′), R1–V2 (5′-GGTGAAAGATCATTCGGCAGAGGAGCG-3′) were used to check for correct chromosomal integration of the *tet* gene.

*Hin*dIII-*Xba*I and *Eco*RI-*Bam*HI DNA fragments corresponding to regions upstream and downstream from the *abrB2* gene were generated from the *B. cereus* chromosome by PCR, with the primers R3–1 (5′-CCC**AAGCTT**GAGGGAGGAAAGATGGAA-3′) R3–2 (5′-GC**TCTAGA**TGCTATCTGCTTTACGAGT-3′) and R3–3 (5′-CG**GAATTC**CAAACGGGATGGAACTG-3′) R3–4 (5′-GC**GGATCC**CACAAGTATAAGGATTATG-3′). External primers (R3–V1 5′-CCGCTATCTATTGTACAACC-3′), R3–V2 (5′-ATCGTGTTCGTCTTCGCCAT-3′) were used to check for correct chromosomal integration of the *tet* gene.

### Plasmid Construction

pHT304*plcRa*’-lacZ, pHT304*abrB2’*-lacZ and pHT304*yrrT’*-lacZ (Table 1) were obtained by inserting the DNA regions upstream (corresponding to the intergenic region) from the Bc ATCC 14579 *plcRa*, *abrB2* and *yrrT* genes between the *Xba*I and *Pst*I cloning sites of pHT304–18Z [23]. The resulting plasmids were then transferred into *B. cereus* by electroporation.

pHT1618KΩPxyl-*papRa* and pHT1618KΩPxyl-*abrB2* containing the promoterless *papRa* gene or the promoterless *abrB2* gene, the *xylR* repressor gene and the inducible promoter of the *xylA* gene, were constructed as follows. The *papRa* gene was amplified by PCR from chromosomal DNA, using primers 5papRaB (5′- CG**GGATCC**TAAAGGGGGATTTATTATGTTC-3′) harbouring a *Bam*HI restriction site and 3papRaE (5′–CG**GAATTC**GAGGTTCAAAAAATCTACTA-3′) harbouring an *Eco*RI restriction site. The *abrB2* gene was amplified by PCR from chromosomal DNA, using primers 5abrB2B (5′-CG**GGATCC**TAAAGGGTGGCATTTTATGA-3′) harbouring a *Bam*HI restriction site and 3abrB2E (5′–CG**GAATTC** AGTTTCACTTTATTTTAAAAG-3′) harbouring an *Eco*RI restriction site. The amplified fragments were inserted downstream P_xylA_ between the *Bam*HI and *Eco*RI cloning sites of pHT1618KΩPxyl (Table 1) [14].

For complementation studies, the *plcRa* gene was inserted into the low-copy number plasmid pHT304 [53] and introduced into the *plcRa* mutant. For complementation of the *plcRa* –deficient mutant harboring pHT304*abrB2’*-*lacZ*, the *plcRa* gene was inserted into pHT1618K (Table 1) [14].

PlcRa was overproduced, using pET28a (Novagen). This plasmid was constructed by inserting an 892 bp *Nco*I-*Xho*I fragment corresponding to the *plcRa* coding sequence. The DNA fragment corresponding to the *plcRa* sequence was generated by PCR, using oligonucleotides PlcRa1 5′-CATG**CCATGG**AATTTAACGATTTGGGT-3′ and PlcRa2 5′-CCG**CTCGAG**TGAGTGTTTTTTATTTTGTAATTC-3′, thus replacing the TAA stop codon by the *Xho*I restriction site. This allows the creation of a translational fusion, adding six C-terminal His residues and placing expression of the gene under the control of a T7 promoter. All constructs mentioned above were checked by sequencing.

### Use of Synthetic Peptide

Cells were cultured at 37°C in LB medium until *t*_0.2_. The culture was then fractionated and synthetic peptide at different concentrations (1 µM, 2 µM, 4 µM, 8 µM, 20 µM) added to one fraction. Incubation was pursued and β-galactosidase activity assayed for each fraction. The peptide CSIPYEY was synthesized, purified by HPLC and identified by mass spectrophotometry by Covalab (France).

### β-galactosidase Assay

β-Galactosidase activity was assayed as described elsewhere [14] All data are means from assays performed in at least three independent experiments, and the means+standard deviations are shown on graphs.

### Isolation of Total RNA

Total RNA was extracted from samples at the indicated time points. Total RNA was collected and isolated by a previously described procedure [28] with minor modifications: for DNA microarray analysis, 20 ml of each culture was harvested and centrifuged, and 2 ml of phenol-based RNA extraction buffer (TRI reagent; Ambion, United Kingdom) was added to the pellet, after which the cells were snap-frozen in liquid nitrogen. For qPCR analysis, 2 ml of each culture was harvested and 1 ml of TRI reagent was added to the cell pellets. RNA quality was assessed with the Agilent 6000 Nano kit in an Agilent 2100 bioanalyzer (Stratagene, Agilent Technologies, France). RIN values were in the 8 to 10 range. Total RNA was purified from two biological replicates for microarray experiments and RT-qPCR analysis.

### Microarray Hybridization and Data Analysis

cDNA synthesis, Cy3/Cy5 labeling and cDNA purification were carried out as previously described by van Schaik *et al.* [28]. Microarray experiments comparing the transcriptomes of the wild-type strain and the *plcRa* deletion mutant were performed with two independent biological duplicates, with Cy3/Cy5 dye-swapping (GEO accession GSE30514). Custom-made Agilent *B. cereus* microarrays were hybridized with 200 ng of labeled cDNA for each sample. The DNA microarrays used in this study were of the 6×18K format [28]. Slides were scanned with an Agilent microarray scanner (G2565BA) and the data were extracted from the microarrays with Agilent Feature Extraction software (version 8.1.1.1). The data extraction procedure included LOWESS normalization of the raw data. The data were further processed as previously described [28], including the use of the web-based VAMPIRE platform [54] with a *P*-value threshold of 0.05. For gene annotation and metabolic routes, we used the PATRIC and KEGG databases.

### RT-quantitative PCR Analysis

We generated cDNA from 1 µg of total RNA with the AffinityScript qPCR cDNA Synthesis kit (Stratagene, Agilent Technologies, France) and random hexamers. We checked cDNA quality with an Agilent 6000 Pico kit, on an Agilent 2100 bioanalyzer (Stratagene, Agilent Technologies, France). We carried out qPCR in triplicate, in a reaction volume of 20 µl containing 500 pg of cDNA, 15 µl of SYBR® Green PCR Master Mix (Applied Biosystems, Courtaboeuf, France) and 300 nM of each gene-specific primer. The primers were designed with Primer Express® (version 2.0), with the following parameters: mean product length of 70 base pairs (bp), mean primer Tm 59°C and mean primer size 20 bp. We generated standard curves for each set of primers, using serial dilutions (four dilutions) of cDNA obtained from total wild-type strain RNA collected at t_2_. We calculated the R^2^ values for these dilution series and the efficiency of each primer set. Amplification was achieved with an ABI®PRISM 7900 (Applied Biosystems), with the following thermal profile: 2 min at 50°C, 10 min at 95°C followed by 40 cycles of 15 s at 95°C and 60 s at 60°C. The specificity of each amplified PCR product was checked by melting curve analysis. Two endogenous controls, 16S and *tufA* [28] from *B. cereus* ATCC 14579 were tested, and *tufA* was found to be the most reliable in our conditions. We therefore normalized the expression levels of the tested genes against those for the *tufA* gene. The relative change in gene expression was recorded as the ratio of normalized target concentrations and was calculated by the comparative ΔΔCt method [55]. RQ Manager (Applied Biosystems) was used to generate expression ratios. The mean values for two independent experiments are presented. Standard deviations were less than 5% of the mean.

### Overproduction and Batch Purification of 6His-tagged-PlcRa

We used pET28a *plcRa* to transform *E.coli* strain BL21. The resulting strain was grown at 30°C in LB with kanamycine 20 µg ml-1 until mid-exponential growth phase (OD_600_ 0.7); IPTG was added (1 mM) and incubation continued for 4 h at 30°C. The cells were centrifuged at 5000 g for 10 min and resuspended in 1/50 of the culture volume of Lysis Buffer (50 mM NaH2PO4 pH 8, 300 mM NaCl, 10 mM imidazole). The cells were incubated during 30 minutes on ice with lysosyme (1 mg/ml) and then disrupted by sonication, and cell debris was removed by centrifugation at 12 000 g for 20 min at 4°C. The resulting crude protein extracts were loaded onto a 0.5 ml Ni-NTA–agarose column (QIAGEN) during one hour at 4°C. After washing, 6His-tagged-PlcRa protein was eluted 4 times with 0.5 ml Elution Buffer (50 mM NaH_2_PO_4_ pH 8, 300 mM NaCl, 250 mM imidazole). Elution samples 2 and 3 were pooled and a Sephadex G-25 buffer exchange colonn was used (Pharmacia) for recovering 6His-tagged-PlcRa protein in Storage Buffer (10 mM Tris, pH7.5, 50 mM KCL, 1 mM DTT). Purified PlcRa aliquots were stored at −70°C. All purification steps were analyzed by SDS–PAGE in a 12% acrylamide gel. The molecular size reference marker was obtained from Bio-Rad. Protein concentrations were determined with the Bio-Rad protein assay.

### Electrophoretic Mobility Shift Assay (EMSA) Assays

A 175-bp DNA probe of the *abrB2* promoter region and a 180-bp DNA probe of the *ilsA* promoter region (negative control) were generated by PCR from BC14579 genomic DNA using 5′ end biotin oligonucleotide primers (Eurofins GENOMICS, LES Ulis, France). For competition assay, a 175-bp DNA probe of the *abrB2* promoter region was generated by PCR from BC14579 genomic DNA using oligonucleotide primers (Eurofins GENOMICS, LES Ulis, France). All PCR fragments were extracted from gels with the QIAquick Gel Extraction Kit (QIAGEN, France) and NanoDrop 2000 spectrophotometer (Thermo scientific) was used for DNA quantification. EMSA experiments were done according to the protocol of LightShift Chemiluminescent EMSA kit from Thermo Fisher Scientific (Brebières, France) and was performed in a 20 µl reaction volume containing 10 mM Tris, pH7.5, 200 mM KCL, 1 mM DTT, 20 µM or 2 µM or 0.2 µM PapRa_7_ and a non specific competitor, 250 ng final salmon sperm DNA. 5 fmol of DNA biotin probe and 200 nM or 1.2 µM or 2 µM of PlcRa were used for each reaction. Competition assay was done with *abrB2* probe at 2.5 pmol. Electrophoresis was performed with non denaturing TBE-acrylamide gels (6%).

### Stress Assays

Viability in the presence of H_2_O_2_ and diamide was assessed in cultures grown in LB medium until the onset of the stationary phase (∼OD 3, ∼*t*_0,4_). The final stock solutions of diamide (1M) or H_2_O_2_ (100 mM) was prepared in sterile demineralized water immediately before use. Cultures were then split in two, with one of the two halves exposed to 1 mM hydrogen peroxide (Sigma) for 10 minutes or 10 mM diamide (Sigma) for 40 minutes. Cells were serially diluted in 0.9% sodium chloride and viability was analyzed by assessing growth on LB agar. We determined the sensitivity of growth to hydrogen peroxide, by culturing cells either in LB medium alone or in LB supplemented with cystine (1 mM) until the end of exponential growth phase growth phase (∼OD 2, ∼*t−*_0,3_). Cultures were split in two, and one half was exposed to 0.4 mM hydrogen peroxide (Sigma). Changes in OD_600_ were monitored until *t*_4_, to monitor growth arrest and estimate the effect of stress.

### Sequence Analysis

Sequences were retrieved with Blast-tp from the NCBI website, http://blast.ncbi.nlm.nih.gov/Blast.cgi, with PLCRa, NP_830774.1 used as the query sequence, the Blosum 62 matrix and all non redundant GenBank CDS translations, PDB, SwissProt, PIR &PRF databases.

### Homology Modeling

Homology modeling of the PlcRa homodimer was performed with Modeler 8v0, using the crystal structure of the complex PlcR/PapR as the template (group I, PDB entry 2QFC). We sequentially generated 30 models of PlcRa chain A and PlcRa chain B satisfying the spatial restraints imposed by the two-dimensional alignment with the target protein. The best model for each chain was selected on the basis of the score function in Modeler [20]. To build the homodimer, the homology model of each chain A and B was then superimposed to its corresponding A and B chains of the target structure using DaliLite from the EBI website (http://www.ebi.ac.uk/DaliLite/). The stereochemistry of the homodimer PlcRa was finally checked using MolProbity (http://molprobity.biochem.duke.edu/). Finally, minor repositionings of side chains were carried out using CHARMm forcefield implemented in Accelrys©. The binding of PapRa_7_ was optimized using CHARMm forcefield.

### Microarray Data Accession Number

The microarray data presented in Tables 2, 3 and in supplementary data, have been deposited in the Gene Expression Omnibus repository (http://www.ncbi.nlm.nih.gov/projects/geo), under accession number GSE30514.

## Supporting Information

Figure S1 **Reconstruction of the sulfur metabolism pathway in** ***B.*** **cereus: transport and biosynthesis of sulfur-containing amino acids.** The putative proteins involved in the uptake and assimilation of inorganic (sulfate) and organic sulfur sources (sulfonates, cystine, methionine) are indicated by the corresponding genes. The BC numbers (ATCC 14579 strain) for *B. cereus* genes are shown, with gene names according to the orthologs in *B. subtilis*. ‘?’ indicates genes probably involved in the pathway or a step for which a gene is lacking or remains to be identified. All the PlcRa-regulated genes involved in sulfur metabolism are indicated by a downward black arrow and the putative functions of all the corresponding proteins are presented in Table 2. Presumed direct targets of CymR are indicated in bold typeface. OAS, *O*-acetyl-serine; AdoMet, S-adenosyl-methionine.
(PPTX)

Figure S2 **Identification of a motif common to the promoter regions of putative CymR targets in the** ***B. cereus*** **ATCC 14579 strain.** An alignment of the promoter regions of the *ssuB*, *yrrT*, *tcyP*, *luxS*, *cysK*, *cysH* and BC1090 genes is presented. The consensus sequence for the CymR-binding site was determined with the WebLogo tool.
(PPTX)

Figure S3 **Kinetics of** ***cymR*** **gene expression.** β-galactosidase specific activity (U/mg protein) of the wild-type **(**black circles), Δ*plcRa* (white triangles) strains harboring the transcriptional P*_cymR_*’-*lacZ* fusion, in LB. Errors bars are shown. Time zero corresponds to the onset of the stationary growth phase, and *t*_n_ is the number of hours before (–) or after time zero.
(PPTX)

Figure S4 **Kinetics of** ***plcRa*** **gene expression.** β-galactosidase specific activity (U/mg protein) of the wild-type strain harboring the transcriptional P*_plcRa_’-lacZ* fusion, in LB medium without or with hydrogen peroxide (0.4 mM) or diamide (10 mM). Hydrogen peroxide or diamide was added at an OD of 2 (∼*t*_−0.3_). Errors bars are shown.
(PPTX)

Table S1 a. Genes with differences of 0.33 fold of less are presented. b. Locus tag in type strain ATCC 14579. c. The gene names indicated correspond to *B. subtilis* homologs, with the exception of *hblB*, *hblL1*, *hblL2* which correspond to gene names in *B. cereus*. *These genes were also analysed with qRT-PCR at t1 and the expression ratio was 0.1.
(DOC)
