# Supplementary material for: PlcRa, a New Quorum-Sensing Regulator from Bacillus cereus, Plays a Role in Oxidative Stress Responses and Cysteine Metabolism in Stationary Phase
Source: PLoS One. 2012 Dec 11;7(12):e51047. doi: 10.1371/journal.pone.0051047 (PMC3519770; doi:10.1371/journal.pone.0051047)
Supplement: Table S1 — a. Genes with differences of 0.33 fold of less are presented. b. Locus tag in type strain ATCC 14579. c. The gene names indicated correspond to B. subtilis homologs, with the exception of hblB, hblL1, hblL2 which correspond to gene names in B. cereus. *These genes were also analysed with qRT-PCR at t1 and the expression ratio was 0.1. (DOC) [file pone.0051047.s005.doc]

**Table S1.** Genes less strongly expressed in the wild-type strain than in the *B. cereus* ∆*plcRa* mutant strain one hour (*t*_1_) and two hours (*t*_2_) after entry into stationary phase, as determined by microarray analysis*^a^*.

|  |  | **Wild type/∆*plcRa* expression ratio expression ratio** | |
| --- | --- | --- | --- |
| **Locus tag***^b^* ***synonym****^c^* | **Function/similarity** | ***t*_1_** | ***t*_2_** |
| *σ^B^ - regulated genes* | |  |  |
| BC0998 *yflT* | General stress protein 17M | 1 | 0.09 |
| BC0999 | Putative stress protein CsbD-like | 1 | 0.08 |
| BC1000 | Hypothetical membrane-spanning protein | 1 | 0.1 |
| BC1002 *rsbV* | Anti-sigma B factor antagonist | 1 | 0.3 |
| BC1003 *rsbW* | Anti-sigma B factor /Serine-protein kinase RsbW | 1 | 0.2 |
| BC1004 *sigB* | RNA polymerase sigma-B factor | 1 | 0.2 |
| BC1005 | Bacterioferritin | 1 | 0.1 |
| BC1006 *rsbP* | Sigma factor sigB regulation protein rsbU | 1 | 0.3 |
| BC1009 | Hypothetical protein | 1 | 0.2 |
| BC3132 | General stress protein 17M | 1 | 0.2 |
| Sub total 10 |  |  |  |
| *Virulence* |  |  |  |
| BC3102 (*hblB)* | Enterotoxin | 0.4 | 0.4 |
| BC3103 (*hblL1)* | Enterotoxin | 0.3 | 0.4 |
| BC3104 (*hblL2)* | Enterotoxin | 0.3 | 0.4 |
| Sub total 3 |  |  |  |
| phBC6A51 *prophage* **region** |  |  |  |
| BC1851 | Putative transcriptional regulator | 0.3 | 1 |
| *BC1852 | Putative chromosomal ATPases SbcC-like | 0.1 | 0.4 |
| *BC1853 | Hypothetical protein | 0.1 | 0.4 |
| *BC1855 | Putative chromosome segregation ATPases | 0.2 | 0.4 |
| *BC1857 | Putative SbcD-like | 0.2 | 0.4 |
| BC1858 | Phage protein | 0.2 | 0.4 |
| BC1859 | Phage protein | 0.3 | 0.4 |
| BC1860 | Phage protein | 0.2 | 0.4 |
| BC1861 | Putative DNA/RNA helicase (DEAD/DEAH box) family) | 0.3 | 0.4 |
| BC1863 | Putative MCM domain family protein | 0.2 | 0.5 |
| BC1864 | Putative DNA polymerase I | 0.3 | 0.5 |
| Sub total 11 |  |  |  |
| phBC6A52 prophage **region** |  |  |  |
| BC2561 | Phage protein | 1 | 0.2 |
| BC2562 | Phage protein | 1 | 0.2 |
| BC2563 | Putative phage replication protein | 1 | 0.3 |
| BC2565 | Phage protein | 1 | 0.3 |
| BC2580 | Phage protein | 1 | 0.2 |
| BC2581 | Putative phage endonuclease | 1 | 0.1 |
| BC2582 | Terminase small subunit | 1 | 0.1 |
| BC2583 | Terminase small subunit | 1 | 0.1 |
| BC2585 | Portal protein | 1 | 0.1 |
| BC2586 | Putative phage prohead protease | 1 | 0.1 |
| BC2587 | Putative phage prohead protease | 1 | 0.1 |
| BC2588 | Phage protein | 1 | 0.1 |
| BC2589 | Phage protein | 1 | 0.1 |
| BC2590 | Phage protein | 1 | 0.1 |
| BC2591 | Phage protein | 1 | 0.1 |
| BC2592 | Phage protein | 1 | 0.1 |
| BC2593 | Phage protein | 1 | 0.1 |
| BC2594 | Phage protein | 1 | 0.1 |
| BC2595 | Gp13 protein | 1 | 0.1 |
| BC2597 | Phage protein | 1 | 0.1 |
| BC2599 | Holin | 1 | 0.3 |
| Sub total 21 |  |  |  |
| *Miscellaneous* |  |  |  |
| BC4314 *hrcA* | Heat-inducible transcription repressor HrcA | 1 | 0.3 |
| BC4140 | Mg(^2+^) transport ATPase. P-type | 0.2 | 1 |
| BC0710 *pstS* | Phosphate-binding protein | 1 | 0.3 |
| BC0711 *pstC* | Phosphate transport system permease protein | 1 | 0.3 |
| BC0712 *pstA* | Phosphate transport system permease protein | 1 | 0.3 |
| BC1793 | Chlorohydrolase/deaminase family protein | 1 | 0.3 |
| BC4343 *phoB* | Alkaline phosphatase | 1 | 0.3 |
| Sub total 7 |  |  |  |
| *Hypothetical proteins* |  |  |  |
| BC0494 | Hypothetical cytosolic protein | 1 | 0.3 |
| BC1132 | Hypothetical protein | 1 | 0.3 |
| BC1151 | IG hypothetical 17028 | 1 | 0.2 |
| BC1176 | Hypothetical protein | 1 | 0.2 |
| BC1805 | Hypothetical protein | 1 | 0.3 |
| BC2612 | Hypothetical protein | 1 | 0.1 |
| BC2994 | Hypothetical protein | 1 | 0.2 |
| BC3610 | Hypothetical protein | 1 | 0.2 |
| BC3965 | Hypothetical protein | 1 | 0.3 |
| BC3966 | Hypothetical protein | 1 | 0.3 |
| BC3985 | Hypothetical cytosolic protein | 1 | 0.3 |
| BC4231 | Hypothetical protein | 1 | 0.3 |
| BC4417 | Hypothetical protein: BofC protein N ter domain | 1 | 0.3 |
| BC4922 | Hypothetical membrane-associated protein | 1 | 0.3 |
| BC4997 | Hypothetical protein | 1 | 0.3 |
| BC4998 | Hypothetical protein | 1 | 0.3 |
| Sub total 16 |  |  |  |
| **Total 68** |  |  |  |

a. Genes with differences of 0.33 fold of less are presented. b. Locus tag in type strain ATCC 14579. C The gene names indicated correspond to *B. subtilis* homologs, with the exception of *hblB*, *hblL1*, *hblL2* which correspond to gene names in *B. cereus*.

*These genes were also analysed with qRT-PCR at *t*_1_ and the expression ratio was 0.1.
